# Antimicrobial Photodynamic Inactivation: An Alternative for Group B *Streptococcus* Vaginal Colonization in a Murine Experimental Model

**DOI:** 10.3390/antiox12040847

**Published:** 2023-04-01

**Authors:** Michał K. Pierański, Jan G. Kosiński, Klaudia Szymczak, Piotr Sadowski, Mariusz Grinholc

**Affiliations:** 1 Laboratory of Photobiology and Molecular Diagnostics, Intercollegiate Faculty of Biotechnology University of Gdańsk and Medical University of Gdańsk, University of Gdańsk, 80-307 Gdańsk, Poland; 2 Department of Computational Biology, Institute of Molecular Biology and Biotechnology, Faculty of Biology, Adam Mickiewicz University in Poznań, 61-712 Poznań, Poland; 3 Department of Pathomorphology, University Hospital in Kraków, 31-501 Kraków, Poland

**Keywords:** *Streptococcus agalactiae*, biofilm, murine model, photoinactivation, rose bengal, vaginal microbiome, metagenomics

## Abstract

Background: *Streptococcus agalactiae*, referred to as Group B *Streptococcus* (GBS), is a prominent bacterium causing life-threatening neonatal infections. Although antibiotics are efficient against GBS, growing antibiotic resistance forces the search for alternative treatments and/or prevention approaches. Antimicrobial photodynamic inactivation (aPDI) appears to be a potent alternative non-antibiotic strategy against GBS. Methods: The effect of rose bengal aPDI on various GBS serotypes, *Lactobacillus* species, human eukaryotic cell lines and microbial vaginal flora composition was evaluated. Results: RB-mediated aPDI was evidenced to exert high bactericidal efficacy towards *S. agalactiae* in vitro (>4 log_10_ units of viability reduction for planktonic and >2 log_10_ units for multispecies biofilm culture) and in vivo (ca. 2 log_10_ units of viability reduction in mice vaginal GBS colonization model) in microbiological and metagenomic analyses. At the same time, RB-mediated aPDI was evidenced to be not mutagenic and safe for human vaginal cells, as well as capable of maintaining the balance and viability of vaginal microbial flora. Conclusions: aPDI can efficiently kill GBS and serve as an alternative approach against GBS vaginal colonization and/or infections.

## 1. Introduction

Group B *Streptococcus* (GBS) is the leading cause of invasive infections among neonates and accounts (together with *Escherichia coli*) for at least 60% of all deaths from neonatal bacterial meningitis [1]. It is a Gram-positive, commensal organism of adult humans’ genitourinary and gastrointestinal tracts, which asymptomatically colonizes the vaginal tract of 15–40% of pregnant women [1]. The most common cause of neonatal GBS disease is maternal colonization with *Streptococcus agalactiae* and its transmission to the fetus and newborns, leading to pneumonia, septicemia and meningitis [2]. The current prevention strategy is intrapartum antibiotic prophylaxis (IAP), applicable for pregnant women at risk of GBS transmission to newborns, which substantially reduces incidences of pneumonia and septicemia; however, the emergence of GBS meningitis, though stable over the past two decades, appears to increase [3,4]. Still, morbidity rates resulting from GBS infection remain unacceptably high, and up to 50% of surviving infants suffer from neurodevelopmental impairment [5]. Moreover, current antibiotic prophylaxis may be characterized by many shortcomings, especially in the case of women who deliver before being screened for GBS colonization and do not comply with the indications regarding the rules for taking or dosing the antibiotic, do not complete antibiotic therapy or are allergic to antibiotics. Obviously, the development of antibiotic resistance is the next important issue. Penicillin-tolerant strains of GBS have been already identified, and resistance to other antibiotics has also been demonstrated [2]. Without alternative prevention strategies, IAP using first-line antibiotics would likely become useless. The issues mentioned above indicate the urgent need for new preventive and/or therapeutic strategies.

Antimicrobial photodynamic inactivation (aPDI) has recently gained increasing importance as a novel, non-antibiotic method targeting a wide variety of both Gram-positive and Gram-negative microbes [6,7,8,9,10]. In principle, the photochemical mechanism of aPDI is based on the excitation of a photosensitive compound by the light of a proper wavelength, resulting in the production of reactive oxygen species (ROS), which lead to damage to the cellular structures that are fundamental to the survival of microorganisms. The formation of singlet oxygen as the most important cytotoxic molecule leads to the oxidation of lipids, proteins and nucleic acids, which results in cell death [11]. In addition to singlet oxygen, other ROS, i.e., hydroxyl radicals, hydrogen peroxide and superoxide anions, were also demonstrated to be formed upon aPDI and to exert bactericidal effects [12]. The most crucial advantage of aPDI that needs to be highlighted is the fact that its bactericidal efficacy is not affected by the antibiotic resistance of the targeted microorganisms. Moreover, no aPDI-resistant microorganisms have been reported [13,14,15].

One of the most important advantages of rose bengal (RB), which was used in this study as a photosensitizer, is its water solubility and low cost. RB is a type II photosensitizer, which means that it can transfer energy directly to ground-state molecular oxygen, which results in the formation of singlet oxygen. RB singlet oxygen quantum yields range between 0.6 and 0.8 [16]. It has both photosensitive and sono-sensitive properties. RB is already approved by FDA as a vital stain to assess the ocular surface. RB was previously reported to be useful in photochemical tissue bonding, photodynamic inactivation (antimicrobial and anticancer), photothrombotic animal models and other applications [17,18,19,20].

In the view of the above data and the antimicrobial properties of aPDI, the current study aimed to assess photodynamic inactivation as an alternative for GBS vaginal colonization. Here, we present an in vitro (planktonic and biofilm culture), in vivo and bioinformatic study demonstrating the high bactericidal efficacy of aPDI towards *S. agalactiae*. The applied treatment was evidenced to be non-mutagenic and safe for human vaginal cells, while also leading to the maintenance of the balance and vitality of vaginal microbial flora.

## 2. Materials and Methods

### 2.1. Bacterial Strains and Culture Media

In this study, 7 strains of *Streptococcus agalactiae* were used, including ATCC 27956 and 6 clinical strains, namely 1030/06, 1029/06, 2306/06, 1153/07, 2974/07 and 3301/08, representing serotypes IA, IB, III, IV, V and IX, respectively. Izabela Sitkiewicz, Translational Medicine Center, Warsaw University of Life Sciences, Warsaw, Poland kindly provided the clinical strains. Additionally, 3 *Lactobacillus* species were included in the analysis, specifically *L. gasseri* LMG13134, *L. crispatus* LMG 12005 and *L. jensenii* LMG 06414, which were purchased from BCCM/LMG. Columbia blood agar plates (GRASO, Starogard Gdanski, Poland) were used for colony-forming unit (CFU) determination. Tryptic soy broth (TSB) (Biomerieux, Craponne, France) was used for overnight planktonic culture of *S. agalactiae* and Schaedler broth (Oxoid, Basingstoke, UK) with a 10% addition of horse serum (Sigma Aldrich, Saint Louis, MO, USA) for *Lactobacilli.*

### 2.2. Photosensitizing Agents

4,5,6,7-Tetrachloro-20-,40-,50-,70- tetraiodofluorescein disodium salt (Rose Bengal, RB) powder was purchased from Sigma (Sigma-Aldrich, Saint Louis, MO, USA). A stock solution (10 mM) was prepared in Millipore distilled water and kept in the dark at 4 °C.

### 2.3. Light Sources

For in vitro studies, a custom-constructed LED-based light source was used, which emitted λ_max_ 522 nm light with a radiosity of 10.6 mW/cm^2^ (FWHM (full-width half-maximum): 34 nm) (Cezos, Gdynia, Poland). For in vivo studies, a custom-constructed LED-based light source, coupled with a spherical light distributor, model SD200 (Medlight S.A., Écublens, Switzerland), was used. This light source emitted λ_max_ 555 nm light with a radiosity of 77.2 mW/cm^2^ at the output and a radiosity of 1.8 mW/cm^2^ with a spherical light distribution (FWHM (full-width half-maximum): 50 nm) (Cezos, Gdynia, Poland).

### 2.4. Photodynamic Inactivation of Planktonic Cultures

Photodynamic inactivation protocol was previously described [21]. In short, overnight cultures of *S. agalactiae* or *Lactobacilli* were adjusted to 2.4 or 2.0 McFarland units, respectively, which corresponds to a cell density of approx. 10^8^ CFU/mL. Bacterial suspensions were mixed with the photosensitizer solution and incubated in the dark at 37 °C for 15 min. Then, samples were washed and resuspended in PBS. Aliquots of 100 µL of each sample were transferred into a 96-well plate and illuminated with a 522 nm LED lamp for 6 min (3.8 J/cm^2^). Afterward, samples were serially diluted in PBS and transferred to Columbia blood agar plates. After 18 h of incubation at 37 °C, the colonies were counted, and CFU/mL values were determined. The experiment was conducted in three replicates.

### 2.5. Microtiter Plate Multispecies Biofilm Culture and Photodynamic Inactivation

Biofilm culture and photodynamic inactivation of biofilm were previously described [21]. In short, biofilms were cultured on 96-well flat-bottom microtiter plates. Overnight *S. agalactiae* culture was concentrated 8 times, *L. jensenii* was adjusted to 2.0 McFarland units (initial bacterial inoculum 10^8^ CFU/mL) and 10 µL aliquots of each bacterium were mixed in a well with 180 µL of Schaedler broth with a 5% addition of horse serum. The plate was covered and incubated at 37 °C and 5% CO_2_ for 4 h. Then, the medium was removed, replaced with 200 µL of fresh medium and incubated at 37 °C and 5% CO_2_ for 20 h. Then, biofilms were incubated with RB, washed and illuminated. After dispersing and serial dilutions, samples were plated on Columbia blood agar plates for *S. agalactiae* enumeration and on Columbia blood agar plates with 4 µg/mL of ciprofloxacin for *L. jensenii*. The experiment was conducted in three replicates.

### 2.6. Photo- and Cytotoxicity Assays Based on MTT

Photo- and cytotoxicity assays were previously described [15]. Briefly, HaCaT cells (CLS 300493) or human vaginal epithelial cells VK2E6E7 (ATCC CRL-2616, Washington, USA) cells were seeded 24 or 48 h before the treatment, respectively, in two 96-well plates (under light and dark conditions). The cells were grown in a humidified incubator at 37 °C and in a 5% CO_2_ atmosphere in supplemented high-glucose DMEM (HaCaT) or Keratinocyte SFM, supplemented with L-glutamine, EGF and BPE (VK2E6E7) (Life Technologies/Thermo Fisher Scientific, Waltham, MA, USA). RB was added directly to the medium and incubated for 15 min at 37 °C. Then, the cells were washed with PBS, and 100 µL of fresh medium was added. Next, the cells were illuminated with the 3.8 J/cm^2^ dose of a 522 nm light. Cell survival was measured after 24 h of incubation at 37 °C by an MTT [3-(4,5-dimethylthiazol-2-yl)-2,5-diphenyltetrazolium bromide] assay. Briefly, 10 µL of MTT solution (12 mM) was added to each well and incubated for 4 h at 37 °C. The cells were then lysed in DMSO (Sigma-Aldrich, Saint Louis, MO, USA), and the absorbance of the formazan was measured at 550 nm using an EnVision plate reader (Perkin Elmer, Waltham, MA, USA). In order to ensure that during the MTT test the absorbance signal came from formazan, not from the RB remaining in the cells, a spectral scan (450–700 nm) using an EnVision plate reader was performed for HaCaT cells treated with 30 µM RB in the dark and then treated with or without MTT prior to lysis in DMSO. The experiment was conducted in three replicates.

### 2.7. Analysis of Real-Time Cell-Growth Dynamics

Real-time cell-growth analysis on the RTCA device (Roche, Basel, Switzerland) was performed as described previously [22]. Briefly, human keratinocytes (HaCaT) were seeded before treatment at a density of 10^4^ cells per well of E-plate (ACEA Biosciences Inc., San Diego, CA, USA). Treatments were performed when cells reached the exponential growth phase, reflected by cell index (CI) = 1.5–2. The RB at a concentration of 0, 5 or 30 μM was added and left for 15 min incubation at 37 °C in the dark. Next, cells were washed with PBS, and the medium was changed. Plates were illuminated with the 3.8 J/cm^2^ dose of 522 nm light or left in the dark for the corresponding time. Plates were placed in the device, where the CI was measured every 10 min until all treated cells reached the plateau phase of growth. The experiment was conducted in three replicates.

### 2.8. Procariotic Mutagenicity Assay–Ames Test

Prokaryotic mutagenicity assay was performed using the commercial kit Ames Penta 2 (Xenometrix, Allschwil, Switzerland) according to the manufacturer’s protocol. Briefly, 16 h overnight cultures of Salmonella Typhimurium [TA98, TA1535] and *Escherichia coli* [uvrA] were diluted in an exposure medium, incubated for 15 min with various concentrations of RB and exposed to the 522 nm light. As positive controls, 2-Nitrofluorene (TA98 and TA1535) and 4-Nitroquinoline-*N*-oxide (*E. coli* uvrA) were added to the cultures to induce mutations. All samples were then incubated for 90 min in an orbital shaker at 37 °C. Afterward, the exposure medium was added to the incubated cultures, and samples were partitioned into the 384-well plates. Then, microplates were covered with adhesive film, incubated for 48 h at 37 °C in the incubator and the number of revertants was assessed. The experiment was conducted in three replicates.

### 2.9. Eucariotic Mutagenicity Assay–Comet Test

Eukaryotic mutagenicity assay was performed using the commercial kit CometAssay (Trevigen, Gaithersburg, MD, USA) according to the manufacturer’s protocol for alkaline comet assay. Briefly, VK2E6E7 cells were seeded 48 h before treatment at a density of 2 × 10^5^ cells per well on a 12-well plate. Cells were treated with RB-mediated aPDI the same way as for the MTT test or incubated for 20 min at 4 °C with 0.1% H_2_O_2_ was used as a positive control. Cells were then detached using the TrypLE Express Enzyme (Thermo Fisher Scientific, Waltham, MA, USA), washed with PBS and resuspended in PBS to obtain 10^5^ cells/mL. Then, cells were mixed with molten LMAgarose and spread on glass slides. After drying, slides were immersed in lysis solution overnight at 4 °C. The next day, slides were incubated in an alkaline unwinding solution for 1 h at 4 °C and alkaline electrophoresis was performed (21 V, 30 min). Slides were washed twice in H_2_O and once in 70% ethanol. After drying, samples were dyed with SYBR Gold and observed under a Nikon Eclipse TE300 Inverted Fluorescence Microscope using a green fluorescence filter. The experiment was performed in 3 independent repetitions. For each repetition, at least 80 comets were analyzed for each group. Comet images were analyzed using CometScore version 2.0 software created by Rex Hoover.

### 2.10. In Vivo Experiments

BALB/c mice (36 animals) were purchased from Charles River laboratories (Saffron Walden, Great Britain, UK). The mice were housed in the Infectious Animal Facility ABSL3 (Malopolska Centre of Biotechnology, Krakow, Poland). Animals were housed (6 per cage) and maintained on a 12 h light–dark cycle with access to water and food ad libitum. Female mice aged 8-weeks old were divided randomly into 6 groups (1. *S. agalactiae* + 555 nm light; 2. *S. agalactiae* + 5 µM RB aPDI; 3. *S. agalactiae* + 30 µM RB aPDI; 4. *S. agalactiae* + 5 µM RB; 5. *S. agalactiae* + 30 µM RB; and 6. *S. agalactiae* only). On the first day, all mice were subcutaneously given β-Estradiol 3-benzoate (0.02 mg/100 µL of sesame oil/mouse) for the induction of the estrus phase. On the second day, all mice were given 10 µL of *S. agalactiae* (10^5^ CFU/mL) vaginally. On the third day, the vaginal wash with PBS (7 × 50 µL) was performed for bacteria enumeration. On the fourth day, illumination of vaginas was performed for group 1. Meanwhile, for groups 2 and 3, RB was provided vaginally to and after 30 min, vaginas were illuminated (50 min, 5.4 J/cm^2^). Similarly, RB was given vaginally to groups 4 and 5, but no illumination was provided, and group 6 was not treated at all. A vaginal wash was performed after 3 h for all groups. Furthermore, on the 5th day, a vaginal wash was performed for all groups. Afterward, mice were euthanized and vaginal tissue with the cervix was taken for analysis.

### 2.11. Microbiological Analysis of In Vivo Experiments

Vaginal washes were divided into two parts: the first was used immediately for microbiological analysis, while the second was frozen for later DNA isolation. Vaginal wash was serially diluted and plated onto the following 12 different culture media: Columbia blood agar (suitable for all aerobic bacteria culture); mannitol salt agar (for *Staphylococcus* culture); CHROMagar Strep B (for *S. agalactiae* enumeration); Edwards agar (for *Streptococcus* culture); Columbia agar with potassium tellurite (enabling *Enterococcus* culture); potato dextrose agar (for fungi and molds); Levine eosin methylene blue agar (for *Enterobacteriaceae* enumeration); CHROMagar Pseudomonas (promoting *Pseudomonas* growth); MRS Agar (for *Lactobacillus* culture); Schaedler blood agar with vit. K (enabling all anaerobic bacteria growth); Schaedler blood agar (dedicated to Gram-positive anaerobic bacteria); and Schaedler blood agar with kanamycin and vancomycin (used for Gram-negative anaerobic bacteria). All plates were purchased from GRASO, Starogard Gdanski, Poland. Plates for the cultivation of anaerobic bacteria were placed into plastic bags with an AnaeroGen sachet (Oxoid/Argenta Sp. Z.o.o., Poznan, Poland) and sealed. All plates were incubated at 37 °C for 48 h.

### 2.12. Preparation of Samples for DNA Sequencing Analysis

A total of 4 mice from group 3 (*S. agalactiae* + 30 µM RB aPDI) were chosen for sequencing library preparation. After defrosting the vaginal wash samples, total DNA was isolated using the DNeasy PowerSoil Pro Kit (Qiagen, Venlo, The Netherlands) according to the manufacturer’s protocol. For sample enrichment, the 16S rRNA region was amplified using PCR. The oligonucleotide primer sequences used in the library preparation step were 5′ AGTTTGATCCTGGCTCAG 3′ (forward) and 5′ AGGCCCGGGAACGTATTCAC 3′ (reverse). PCR was carried out using Taq polymerase according to the manufacturer’s protocol (Thermo Scientific™, Kansas City, Kansas, USA) for 5 min at 94 °C and 40 cycles of 1 min at 94 °C, 1 min at 55 °C and 2 min at 72 °C, followed by 10 min at 72 °C. Amplification products were then purified using the Syngen PCR Mini Kit (Syngen, Wroclaw, Poland) and sequenced by Genomed (Genomed S. A., Warszawa, Poland). Amplicons of the V3–V4 fragment of the 16S rRNA gene were sequenced using the Illumina MiSeq platform in paired-end mode with two readings of 300 bases [PE 300], assuming min. 50,000 pairs of reads per sample.

### 2.13. Analysis of Sequencing Data

The quality of raw sequencing reads was assessed using FastQC (version 0.11.9) [23] and MultiQC (version 1.14) [24]. Afterward, reads were imported into Qiime2 software (version 2022.11) [25]. However, due to a significant drop in quality of the 3′ ends, only the first 240 and 200 nucleotides from the 5′ ends were selected from the forward and reverse reads, respectively, and were subject to further analysis.

Preprocessed reads were merged, denoised, dereplicated and filtered using the Qiime2 implementation of the dada2 algorithm [26] with default settings. The filtering step was designed to remove low-quality reads and chimera sequences. All the steps were performed using the Qiime2 dada2 module, resulting in the amplicon sequence variants (ASV) observed in the analyzed samples.

Taxonomic classification of the ASVs was accomplished using a BLAST^+^-based classifier [27] called classify-consensus-blast, available in the feature-classifier module [28]. BLAST querying was performed against the QIIME2-compatible version of the SILVA database (release 138) [29,30] containing full-length 16S rRNA sequences preprocessed with RESCRIPt [31] and downloaded from the QIIME2 website (https://docs.qiime2.org/, accessed on 24 January 2023). Relative frequencies of identified taxa abundances are presented in the Supplementary Materials—file (Table S1): rel_freq.xlsx.

Additionally, LEfSe [32] analysis was employed to find significant differences in taxa abundances between D3, D4 and D5. LEfSe (version 1.0) was run using the Galaxy platform [33]. LDA scores were calculated using thresholds set to an alpha value of 0.05 and a logarithmic LDA score for discriminative features of 2. Furthermore, the “strategy for multi-class analyses” option was set to “One against all”.

### 2.14. Histopathological Analysis

Tissues suspended in 10% buffered formalin solution were sent to the Department of Pathomorphology, University Hospital, Krakow. After taking representative sections, the material was dehydrated in a series of increasing ethanol concentrations, cleared in xylene and impregnated with liquid paraffin in a vacuum tissue processor. After embedding in a paraffin block, microscopic preparations were made. The blocks were cut on a rotary microtome at a thickness of 2.5 µm. After drying, slides were stained with hematoxylin and eosin using an automated staining system. Histopathological analysis of the specimens was performed by an experienced pathologist (P.S.) who did not know the status of the examined material. Measurements were made using an Olympus BX53 microscope equipped with Olympus SWH10X-H eyepieces number 26.5 and an Olympus UPlanFLN 40×/0.75 objective. Three measurements were taken, specifically the intensity of the inflammatory infiltration in the proximal 2/3 of the vagina, the distal 1/3, and the degree of neutrophil involvement of the vaginal epithelium. The intensity measurement results were assessed semi-quantitatively using the following scale: 0—none; 0.5—minimum intensity; 1—very weak intensity, 1.5—weak intensity; 2—moderate intensity; 2.5—moderately strong intensity; and 3—strong intensity. In addition, the amount of purulent content within the vaginal lumen was measured. The results of the intensity of purulent content measurements within the vaginal lumen were evaluated semi-quantitatively using the following scale: 0—none; 1—moderately abundant purulent content; and 2—very abundant purulent content.

### 2.15. Statistical Analysis

GraphPad Prism version 9.5.0 for Windows (GraphPad Software, Inc., San Diego, CA, USA) was used to perform statistical analysis. Quantitative variables were characterized by the arithmetic mean and the standard deviation of the mean. Quantitative variables were characterized by the arithmetic mean and the standard deviation of the mean. One-way ANOVA followed by Dunnett’s multiple-comparisons test was used for data analysis. A *p* value of <0.05 was determined as an indicator of a significant difference.

## 3. Results

### 3.1. Streptococcus Agalactiae Can Be Eradicated with RB-Mediated aPDI in Planktonic Culture In Vitro

**Figure 1:**
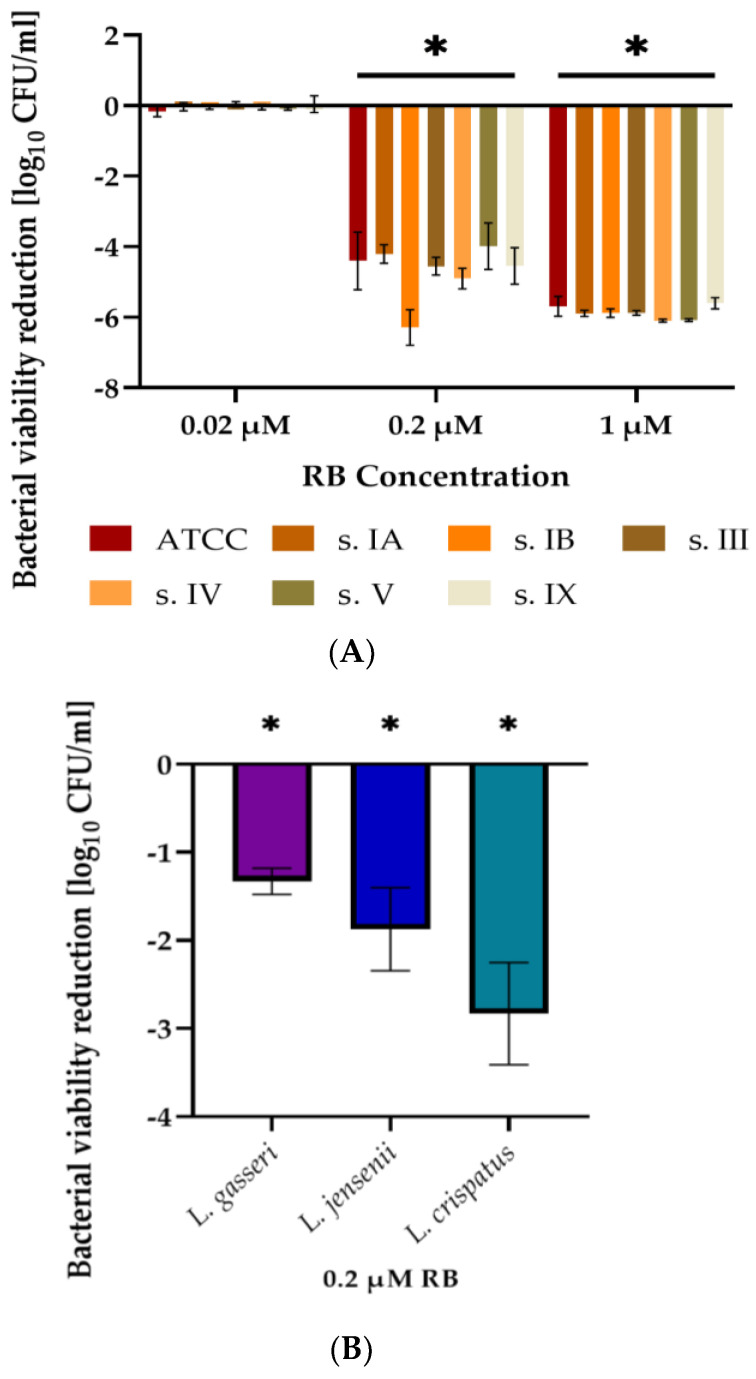

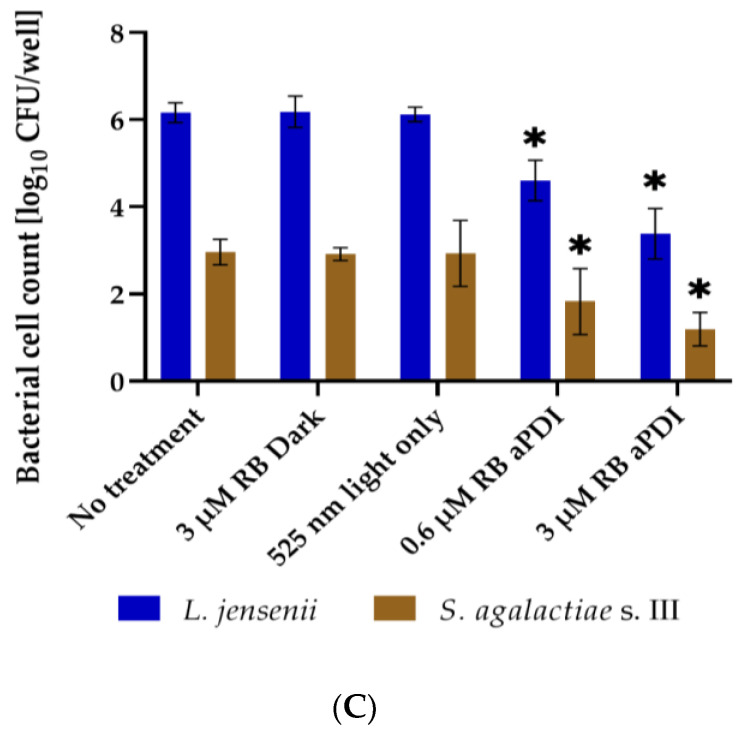
Effect of RB-mediated aPDI on: (**A**) planktonic culture of *S. agalactiae*; (**B**) planktonic culture of human vaginal *Lactobacilli*; (**C**) multispecies biofilm culture of *S. agalactiae* and *L. jensenii.* Significance at the respective *p*-values is marked with asterisk (* *p* < 0.05) with respect to the “No treatment” group.

To verify if RB-mediated aPDI has potential in *S. agalactiae* eradication, the optimization of RB concentration and light dose had to be performed. The main goal was to obtain a lethal dose of aPDI, i.e., viability reduction by >3 log_10_ CFU/mL. These parameters were chosen according to the previous experiments [21] using the ATCC strain. Figure 1A presents viability reduction in *S. agalactiae* caused by aPDI with different RB concentrations for the ATTC strain and six clinical isolates representing six different serotypes. aPDI with 0.2 µM RB was assessed as a lethal dose as it caused 4–6.3 log_10_ units of viability reduction, while aPDI with 1 µM RB caused complete eradication of all strains in vitro.

### 3.2. RB-Mediated aPDI Is Less Effective against Human Vaginal Lactobacilli Than against S. agalactiae In Vitro

Three representatives of the most common vaginal *Lactobacillus* species were chosen to assess the aPDI effect on human physiological vaginal flora. The viability reduction caused by aPDI with 0.2 µM RB was lower for all three *Lactobacillus* species than for *S. agalactiae* and varied between 1.3 to 2.8 log_10_ units (Figure 1B).

### 3.3. RB-Mediated aPDI Inactivates S. agalactiae in Multispecies Biofilm Culture

The culture of multispecies biofilms of *S. agalactiae* and vaginal *Lactobacillus* is presented in Figure 1C. Serotype III of *S. agalactiae* was chosen as a strong biofilm producer. Multispecies biofilms were cultured for *S. agalactiae* with *L. gasseri, L. crispatus* or *L. jensenii*. For each combination, both species were inoculated in the same initial cell number. After 24 h of biofilm culture, fewer *S. agalactiae* cells were observed than *Lactobacillus* cells. Only multispecies biofilms of *S. agalactiae* and *L. jensenii* were presented as representative. As expected, biofilm culture requires use of a much higher concentration of RB than used for planktonic culture. To obtain viability reduction close to the detection limit for *S. agalactiae,* 3 µM RB was used for aPDI. The effectiveness (viability reduction) of aPDI was similar for both species in this multispecies biofilm. However, as for *L. jensenii* the initial cell number was much higher than for *S. agalactiae*; there was still a high number of viable *Lactobacillus* cells after photoinactivation.

### 3.4. RB-Mediated aPDI with Low Concentrations of RB Is Not Toxic for Eukaryotic Cells

**Figure 2:**
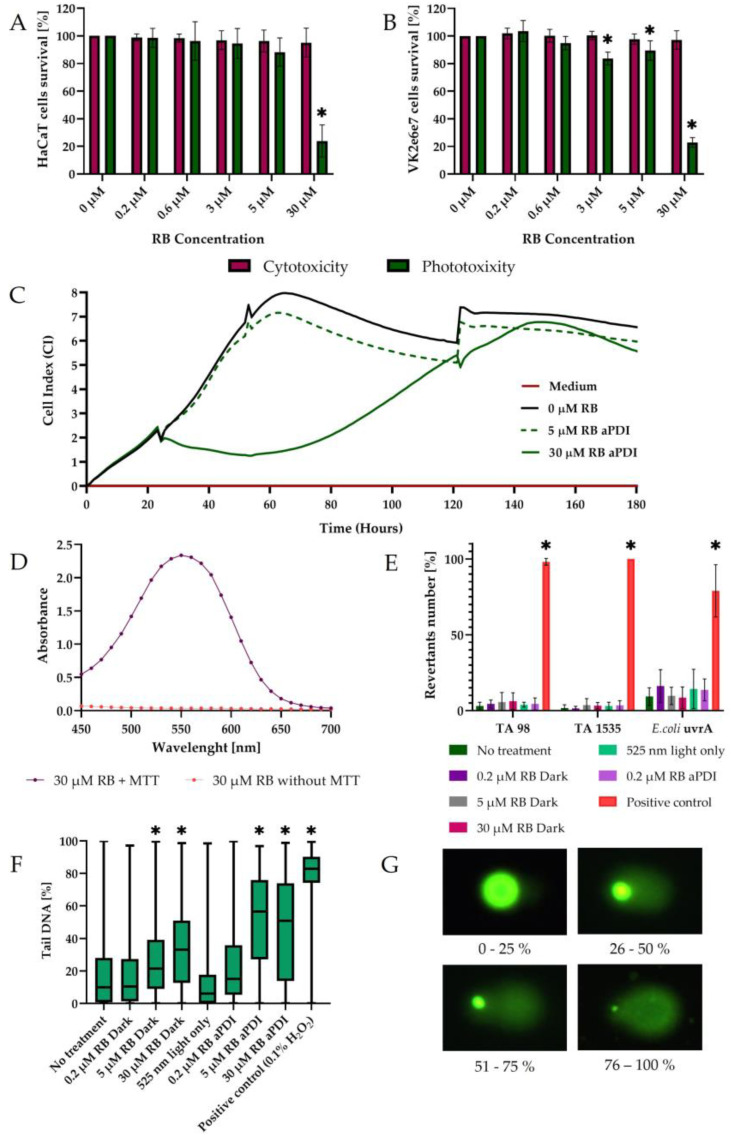
Analysis of RB-mediated aPDI toxicity: (**A**) Effect on human keratinocytes (HaCaT) with MTT test; (**B**) Effect on vaginal epithelial cells (VK2E6E7) with MTT test; (**C**) Effect on real-time Cell-growth dynamics (HaCaT); (**D**) Absorbance spectrum measurement of HaCaT cells after MTT test; (**E**) Mutagenic effect on procaryotic cells via Ames test; (**F**) Mutagenic effect on eucaryotic cells (VK2E6E7) via comet assay; (**G**) Examples of obtained comets in comet assay with different percentage of DNA in comet tail, 100× magnification. Significance at the respective *p*-values is marked with asterisk (* *p* < 0.05) with respect to the “No treatment” group.

To assess the RB-mediated aPDI effect on eucaryotic cells, the MTT test was performed. Human keratinocytes (HaCaT) (Figure 2A) and human vaginal epithelial cells (VK2E6E7) (Figure 2B) were used to investigate the cytotoxicity and phototoxicity of different RB concentrations used for in vitro (0.2, 0.6, 3 µM) and in vivo (5, 30 µM) studies. No cytotoxic effect was observed for any of the above concentrations. Except for 30 µM RB, no concentration caused any significant phototoxicity for HaCaT cells. For VK2E6E7 cells, a small phototoxic effect (<20%) was observed for 3 and 5 µM RB and significant phototoxicity was observed for 30 µM RB, similar to HaCaT cells. Because the MTT test assesses cell viability at only one timepoint (24 h post-treatment), for the highest RB concentrations, real-time cell-growth dynamics of HaCaT cells were also evaluated (Figure 2C). For 5 µM RB aPDI, a slight delay in growth curve was observed in comparison to the control (0 µM RB, dark). However, both groups reached a plateau at a similar time (ca. 60 h from seeding). For cells treated with 30 µM RB aPDI, a decrease in viability was observed shortly after photoinactivation, which is consistent with previous MTT results. Although proliferation was delayed, the cells reached a plateau about 150 h after seeding. This indicates that despite the fact that 30 µM RB aPDI exhibits some phototoxicity, the cells still manage to grow and divide to reach the plateau phase. In order to ensure that during the MTT test, the absorbance signal comes from formazan and not from the RB remaining in the cells, a spectral scan was performed (Figure 2D). It revealed that at 550 nm, the signal from cells treated with RB without MTT is at the background level.

### 3.5. RB-Mediated aPDI in the Ames Test Does Not Show Mutagenicity in Procaryotic Cells

Two strains of Salmonella Typhimurium [TA98, TA1535] and one strain of *Escherichia coli* [uvrA] were used to assess the mutagenicity of RB and RB-mediated aPDI in the Ames test. These strains possess mutations that do not them to grow in the medium with a minimal amount of histidine. When mutations occur, revertant cells are able to grow in a medium with a minimal amount of histidine, which allows us to assess the mutagenicity of the tested compound. Strong mutagens specific for each strain were used as a positive control. In Figure 2E, it can be observed that none of the tested conditions showed significant mutagenicity for any of the tested strains. aDPI of 5 and 30 µM RB had a lethal effect on all tested strains. Consequently, the assessment of these conditions’ mutagenicity in the procaryotic model was not possible.

### 3.6. RB-Mediated aPDI in Comet Assay Does Not Show High Mutagenicity in Eucaryotic Cells

VK2E6E7 cells were used to assess RB-mediated aPDI mutagenicity in eukaryotic cells. After treatment, cells were mixed with agarose and dried on glass slides and alkaline electrophoresis was performed. In this process, DNA from cells containing DNA breaks (as a result of mutagenicity) was able to migrate from the nucleus and form comet tails, which were possible to observe under the fluorescence microscope after DNA staining. In Figure 2G, examples of obtained comets with different percentages of DNA in comet tails are presented. Figure 2F shows the mean percentage of DNA in comet tails for different experimental groups. Light only, 0.2 µM RB and 0.2 µM RB aPDI did not display any mutagenicity compared to the not treated group. Groups 5 and 30 µM RB in the dark showed a slight increase in tail DNA percentage. Groups 5 and 30 µM RB aPDI exhibited moderate mutagenic effects in eucaryotic cells; however, this effect is still much lower than for the positive control (0.1% H_2_O_2_).

### 3.7. RB-Mediated aPDI Can Inactivate S. agalactiae In Vivo in the Murine Model

**Figure 3:**
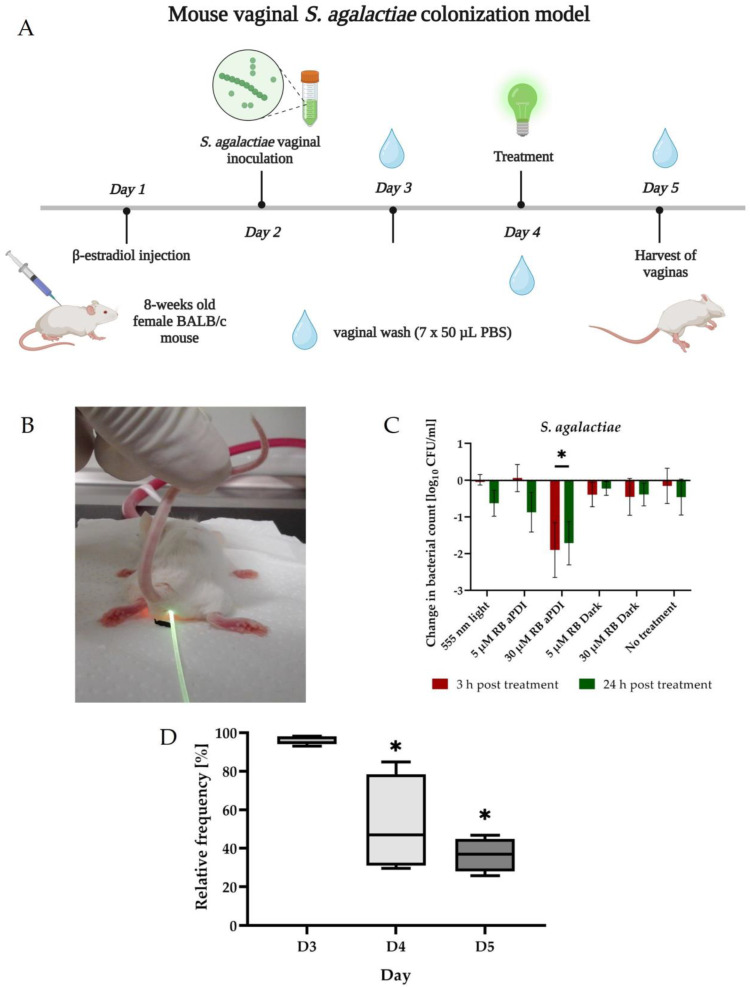
In vivo studies of *S. agalactiae* eradication efficacy with RB-mediated aPDI on murine model: (**A**) Scheme of mouse vaginal *S. agalactiae* colonization model, created with BioRender.com; (**B**) Irradiation of mouse vagina with spherical light distributor; (**C**) Change in *S. agalactiae* bacterial count obtained using microbiological approach in murine model; (**D**) *S. agalactiae* relative abundance in mice treated with 30 µM RB aPDI obtained with metagenomic analysis. Significance at the respective *p*-values is marked with asterisk (* *p* < 0.05) with respect to the “No treatment” group, or with respect to the day 3 group in Figure 3D.

To assess the efficacy of RB-mediated aPDI in vivo, the mouse model of vaginal colonization with *S. agalactiae* was used. The estrus phase was induced in BALB/c mice with β-estradiol. Then, their vaginas were inoculated with *S. agalactiae* (Figure 3A). Vaginal wash was performed the day after inoculation and 3 and 24 h post-treatment. Mice were treated with light only, RB only or RB-mediated aPDI, or they were not treated at all. Figure 3B explains how the vagina was irradiated with a spherical light distributor while the mouse was sedated. The presence of *S. agalactiae* in the vaginal wash was assessed with a microbiological approach, i.e., plating on CHROMagar Strep B (Figure 3C) with a metagenomic approach, as well as isolation and sequencing of DNA (Figure 3D). In the microbiological approach, all tested groups were investigated. For all control groups (no treatment, light only, RB in the dark), a slight reduction in *S. agalactiae* presence in the vagina was observed. For the 5 µM RB aPDI group, the change in *S. agalactiae* presence was comparable to the control groups. In the group treated with 30 µM RB aPDI, the reduction in *S. agalactiae* viability was the highest and reached ca. 2 log_10_ units both 3 and 24 h post-treatment on day four and day five of the experiment, respectively (Figure 3C). For the metagenomic approach, washes from four mice from the group treated with 30 µM RB aPDI were sequenced. The relative frequency of *S. agalactiae* significantly decreased on days four (3 h post-treatment) and five (24 h post-treatment) (Figure 3D). Therefore, this confirms the results obtained using the microbiological approach.

### 3.8. The Histopathological Analysis Does Not Indicate the Negative Impact of RB-Mediated aPDI

**Figure 4:**
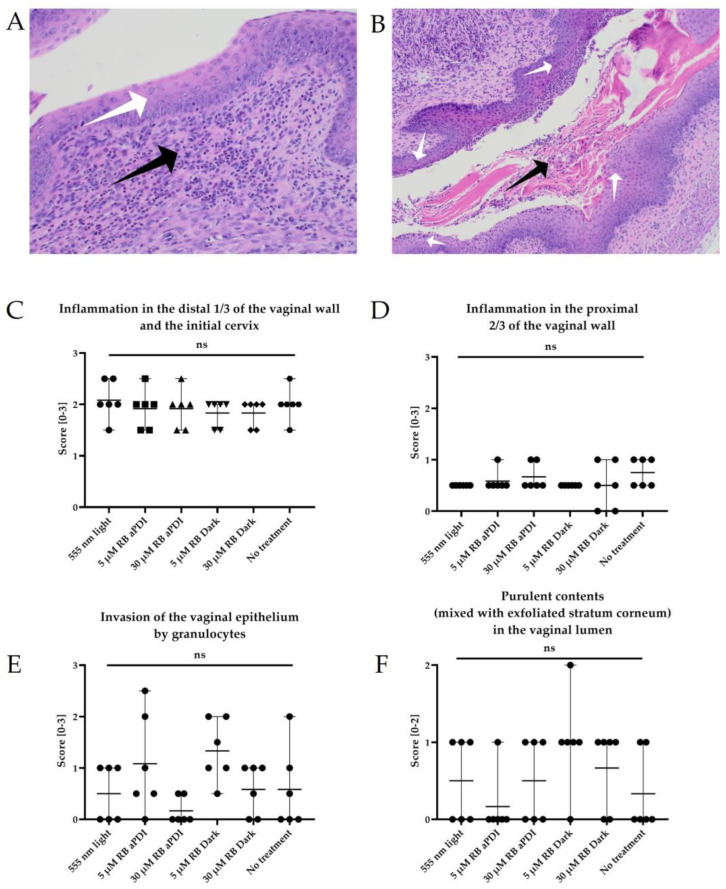
In vivo studies on *S. agalactiae* eradication efficacy with RB-mediated aPDI on murine model via histopathological analysis: (**A**) The distal 1/3 of the vaginal wall, white arrow: stratified squamous epithelium of the vaginal mucosa; black arrow: inflammatory infiltration focus, 200× magnification; (**B**) The distal 1/3 of the vaginal wall, black arrow: purulent content within the lumen of the vagina; white arrows: foci of invasion of the vaginal paraepidermoid epithelium by granulocytes, 100× magnification; (**C**) Inflammation score in the distal 1/3 of the vaginal wall; (**D**) Inflammation score in the proximal 2/3 of the vaginal wall; (**E**) Score of invasion of epithelium by granulocytes; (**F**) Score of purulent content in vaginal lumen. ns, not significant.

After a vaginal wash on day five, all mice were euthanized and vaginal tissues with cervix were collected for histopathological analysis. Slides were searched for inflammation (Figure 4A), purulent content and invasion of epithelium by granulocytes (Figure 4B). Inflammation was assessed in the distal 1/3 of the vaginal wall and in 2/3 proximal of the vaginal wall (Figure 4C,D). No significant differences were observed in this parameter. However, as the invasion of epithelium by granulocytes was evaluated, although with no statistical significance, the score was the lowest for the group treated with 30 µM RB aPDI (Figure 4E). This may indicate that aPDI has a local negative impact on granulocytes and decreases the number of granulocytes on the site of treatment. For the last evaluated parameter, purulent content (mixed with exfoliated stratum corneum), in the vaginal lumen, no significant differences were observed (Figure 4F).

### 3.9. S. agalactiae Colonization Affects the Vaginal Microbiome Composition

**Figure 5:**
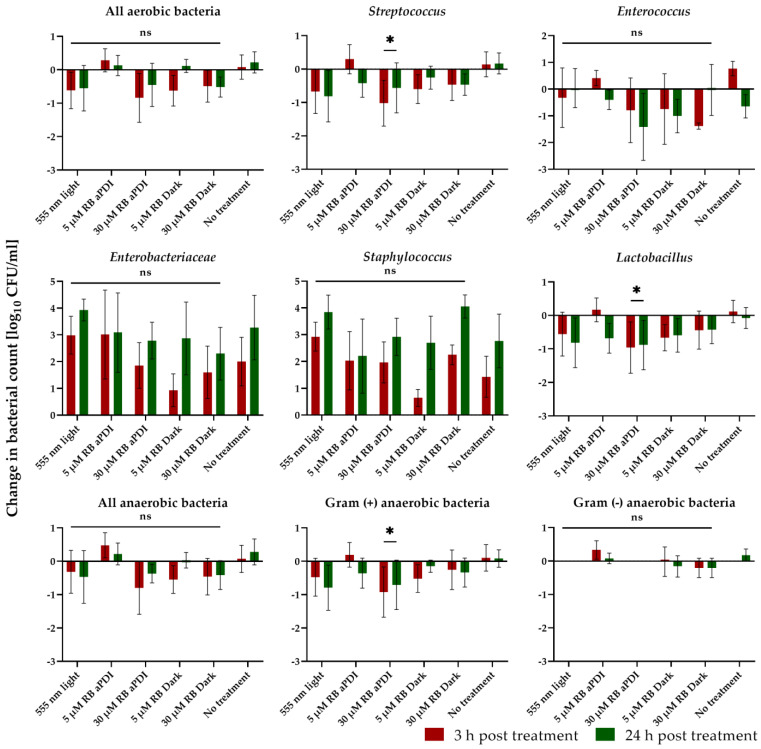
Changes in the bacterial counts obtained using the microbiological approach in a murine model. Significance at the respective *p*-values is marked with asterisk (* *p* < 0.05) with respect to the “No treatment” group; ns, not significant.

To assess the impact of RB-mediated aPDI on vaginal flora, vaginal wash samples were serially diluted and spread on 12 different media. No colonies were observed on plates intended for the cultivation of Pseudomonas or fungi and molds. Changes in *S. agalactiae* growth are described above (Figure 3C). Changes in growth in the rest the groups are presented in Figure 5. Counts of Gram-negative anaerobic bacteria did not change significantly during the experiment. The number of all aerobic bacteria, *Streptococcus, Enterococcus*, and *Lactobacillus*, as well as all anaerobic bacteria and Gram-positive anaerobic bacteria, changed similarly during the experiment. All of these bacterial group counts slightly decreased in most of the treatment groups compared to the non-treated group. Although for *Streptococcus, Lactobacillus* and Gram-positive anaerobic bacteria, changes in bacterial counts were significantly different for 30 µM RB aPDI in comparison to the non-treated group, these changes were lower than in *S. agalactiae* (Figure 3C). The most noticeable change was observed in *Enterobacteriaceae* and *Staphylococcus*. The counts of these two groups increased significantly in all tested groups of mice with no significant differences between groups.

### 3.10. aPDI Impacts the Abundance of the Most Prevalent Species in Mouse Vaginas

**Figure 6:**
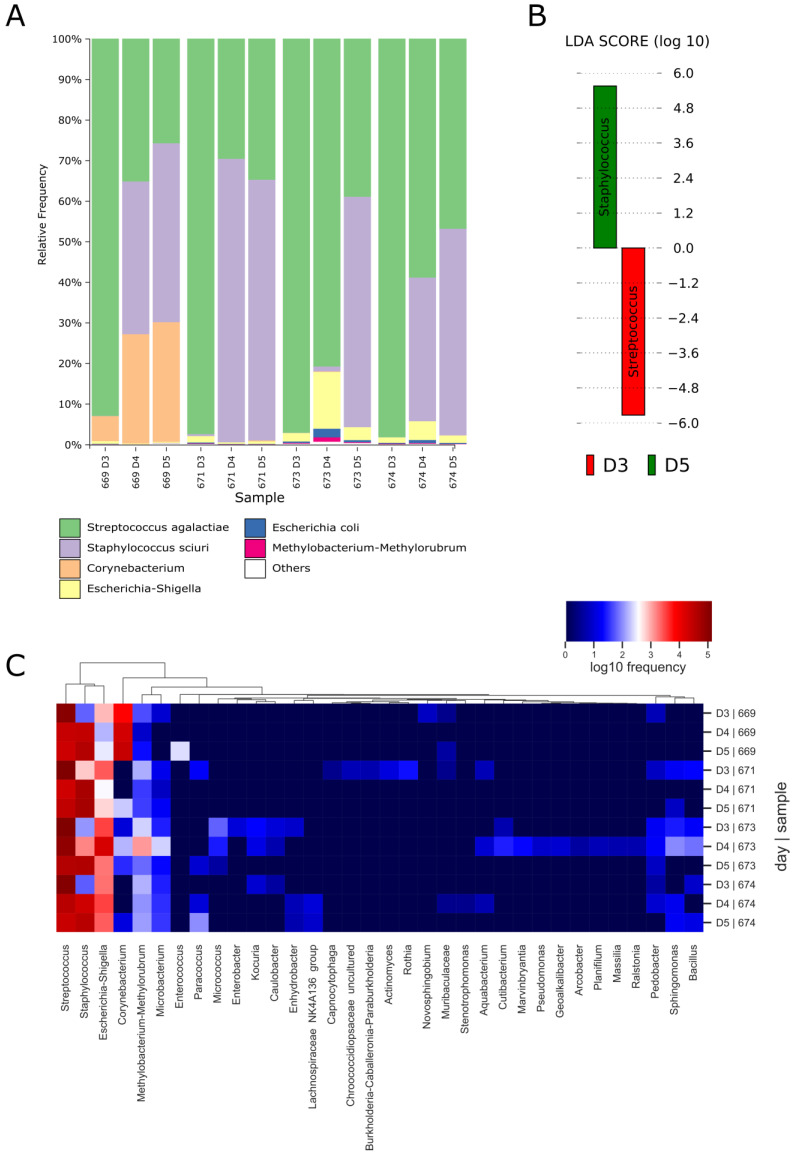
Metagenomic analysis of V3-V4 fragment of 16S rRNA gene sequencing from vaginal wash obtained from mice treated with 30 µM RB aPDI: (**A**) Relative frequency of most abundant bacterial species. Numbers 669, 671, 673 and 674 represent four different mice, while D3, D4, and D5 abbreviations correspond to the day of the experiment; (**B**) Results of LEfSe analysis representing significant differences in taxa abundances between D3, D4 and D5; (**C**) The genus level abundance of vaginal microbiota in each mouse on different days.

As a result of the 16S rRNA gene-sequencing data analysis, we observed the significant impact of aPDI on the microbial communities of the mouse vaginas. The most striking effect was observed in the *S. agalactiae* abundance, during which D3 in all the biological replicates corresponded to more than 90% of identified amplicon sequence variants (ASVs). The relative abundance (Figure 6A,C) of *S. agalactiae* decreased more than two-fold from D3 to D4 and D5, ultimately reaching lows of ~25–45%. In contrast, the relative frequency of *Staphylococcus sciuri* increased at D4 and D5. *S. sciuri* represented less than 1% of all ASVs on D3, while the prevalence of the species increased significantly to ~1–64% on D4, finally reaching 44–64% on D5. To a lesser extent, we observed a significant increase in the relative abundance of *Corynebacterium* from D3 (~6%) to D4 (~27%) and D5 (~29%) in the mice designated 669. In the remaining samples, the share of *Corynebacterium* ASVs did not exceed 1%. Furthermore, the prevalence of the *Escherichia-Shigella* group changed between D3 (up to ~2%), D4 (up to ~16%) and D5 (up to ~3%). Additionally, the presence of *Escherichia coli* (D3: <1%, D4: <1%~2%, D5: <1%~5%) and *Methylobacterium-Methylorubrum* (D3: <1%, D4: <1%~1%, D5: <1%) groups are worthy of mention.

LEfSe analysis (Figure 6B) was performed to determine the most significant differences in taxon abundance between the subsequent days of in vivo experiment. The study has shown that the decreasing trend in *Streptococcus* and the increasing trend in *Staphylococcus* genera abundances between D3 and D5 were statistically significant in terms of the LDA scores shown in Figure 6B.

Further analysis of the heatmap displaying the relative abundances of the observed genera (Figure 6C) suggests that vaginal microbiome composition is similar between analyzed mice. The most significant differences in frequency were observed in *Streptococcus, Staphylococcus, Escherichia-Shigella, Corynebacterium, Methylobacterium-Methylorubrum,* and *Microbacterium* genera.

## 4. Discussion

Recently, several alternatives to current IAP, aiming to prevent or limit maternal GBS colonization, have been explored. These, first of all, include strategies that assume applying compounds with specific antimicrobial activity toward GBS, i.e., plant-derived crude extracts and phytochemicals [34] and plant-based lipids from *Aristolochia longa* and *Bryonia dioica* [35], octylglycerol [36] or synthetic peptides mimicking human C5a, which was demonstrated in in vivo mouse vaginal colonization models to be bactericidal toward GBS [37]. Although these compounds reveal some efficacy against GBS in animal models, it is still to be established if any of them are suitable for human use. As a next alternative to antibiotic treatment, intrapartum chlorhexidine vaginal washes have been considered and confirmed to significantly lower neonatal colonization [38]. Finally, the use of probiotic agents limiting pathogen overgrowth and promoting native vaginal flora has been proposed [39,40]. The most promising probiotic candidates demonstrating beneficiary activity against GBS include the *Lactobacillus* genus, i.e., *L. rhamnosus*, *L. gasseri* or *L. reuteri*.

Here, we describe the use of novel antimicrobial photodynamic inactivation as an attractive and highly effective antimicrobial approach that could potentially lead to control of GBS vaginal colonization with respect to the harmless effect on host cells and the mild influence on viability and composition of vaginal flora. Regardless of the abundant literature regarding the use of aPDI against human microbial pathogens (most recently reviewed by Nakonieczna et al. (2019) [7]), there is only one single report by Sellera et al. (2016) that demonstrates the efficacy of classical aPDI in the inactivation of pathogens associated with bovine mastitis (*S. aureus, Streptococcus agalactiae, S. dysgalactiae, Corynebacterium bovis*, and the alga *Prototheca zopfii*), indicating that aPDI could be an interesting tool for inactivating *S. agalactiae* as a source of veterinary infections [41]. Nevertheless, no studies have yet described the use of aPDI against *S. agalactiae*, which is involved in the etiology of neonatal infections. Moreover, no studies have demonstrated the potential of using aPDI to control GBS vaginal colonization, which emphasizes the novelty of this investigation.

The current study describes aPDI employing a harmless photoactive compound, i.e., rose bengal (RB), which was used in our previous mechanistic studies concerning aPDI treatment [21,42,43]. RB is a xanthene dye, which has been widely applied as a photoactive compound in photodynamic treatment [15,44,45,46,47,48,49,50,51]. Among xanthene dyes, which are potent producers of ROS, such as singlet oxygen [52], RB is one of the most efficient and widely used source of ^1^O_2_ in polar solvents, such as water [53]. Moreover, it is considered a molecule with high biocompatibility (necessary criteria for therapeutic agent) and a low level of cytotoxicity, even at higher concentrations [15,48]. Although most bacteria have advanced systems for ROS neutralization and repair of ROS-induced damage, there are no adequate defenses for singlet oxygen and hydroxyl radicals, so even small amounts of these may be fatal [54]. We have previously shown that *S. agalactiae* responds to aPDI-induced oxidative stress with increased expression of major oxidative stress response elements, such as *sodA*, *ahpC*, *npx*, *cylE*, *tpx* or *recA*. Moreover, even though we observed increased photoinactivation stress tolerance after consecutive treatments in *S. agalactiae*, a slight increase in RB concentration was sufficient to eradicate tolerant strains [43].

The results of the current study demonstrate that various pathogenic GBS serotypes were susceptible to the action of aPDI both in planktonic and multispecies biofilm culture while leaving high viability of main representatives of human vaginal flora, i.e., *Lactobacillus* species. As far as GBS biofilm development appears to support vaginal colonization [55] by effective protection from antimicrobials and host defense factors, the results concerning effective inactivation activity of aPDI toward *S. agalactiae* growing in multispecies biofilm culture should be considered to be of high importance. The human vaginal flora was clustered into five different groups and four of them were evidenced to be dominated by *Lactobacillus* species [56]; thus, three different representatives of *Lactobacillus*, i.e., *L. gasseri*, *L. jensenii* and *L. crispatus* were selected for in vitro evaluation within the current study, indicating that employed aPDI could indeed lead to reduced viability of *Lactobacilli*. However, the remaining portion of these microorganisms is still a two-order magnitude higher than targeted GBS, which enables the efficient recovery of the native vaginal flora.

When searching for alternative prevention and/or treatment approaches, the safety aspects of the strategy must be taken into account. Described aPDI was evidenced to be safe toward both human keratinocytes and vaginal epithelial cells regardless of cyto- and phototoxicity effects. Real-time growth dynamics confirmed that even when high RB concentrations were used (leading to eukaryotic cells inactivation by approximately 80%), treated human cells were capable of efficient recovery upon treatment. Moreover, the proposed approach under studied conditions was evidenced to exert no mutagenic effect (emphasized with the application of FDA-approved Ames methodology using three various microbial mutants) and mild genotoxic effect toward eukaryotic cell lines (much below the positive, genotoxic control). Overall, aPDI safety was also evidenced with the presented mice colonization model via histopathological analysis. Vaginal tissues with the cervix were collected and searched for inflammation, purulent content and invasion of epithelium by granulocytes. No significant differences were observed in these parameters, indicating the safe use of aPDI and exerting no aPDI-induced inflammation.

Finally, aPDI was assessed regarding its influence on the composition and viability of native vaginal flora. Employing a microbiological and bioinformatic approach, we have undoubtedly confirmed that aPDI leads to significant reduction in GBS load within the vaginal tract and the mild inactivation of all (except *Enterobacteriaceae* and *Staphylococcus* genus) microbial groups, i.e., aerobic (including *Streptococcus*, *Enterococcus*, *Lactobacillus*) and anaerobic bacteria (including Gram-positive and Gram-negative bacteria). In the case of *Enterobacteriaceae* and *Staphylococcus*, the increased load of these microbes was reported during the experiment; however, this effect was not dependent on the treatment used and was also observed for the not treated animals. Observed with bioinformatical analysis, *S. sciuri,* although considered pathogenic, commonly occurs in rodents [57,58] and can even have a positive effect, such as preventing asthma in mice [59]. The most probable cause for *Enterobacteriaceae* and *Staphylococcus* prevalence increase is the effect of *S. agalactiae* infection. A similar effect was previously observed in mice vaginas after HSV-2 infection [60]. Vaginal flora of mice in contrast to human vaginal flora is not dominated by *Lactobacilli* [61]. Therefore, it is not possible to predict outcome of aPDI in human vaginal flora based on the results from aPDI treatment of mouse vagina. This investigation is yet to be conducted. There is a possibility that the use of intravaginal probiotic treatment right after aPDI would prevent secondary infections of the urogenital tract.

## 5. Conclusions

In recent decades, GBS has remained a prominent concern for neonatal health. Although IAP has substantially reduced incidence of GBS infections, maternal and infant colonization rates remain a concern in modern medicine. The emergence of novel and beyond broad-spectrum antibiotic alternative strategies to control GBS vaginal colonization appears to be promising for the urgent need to prevent neonatal GBS infections. The present study showed that antimicrobial photodynamic inactivation may serve as a safe alternative option against GBS. Moreover, the global scenario of antibacterial resistance has been of great concern for public health, and novel non-antibiotic strategies could help us resolve this problem. Obviously, further detailed, preclinical studies assessing the bactericidal efficacy and toxicological aspects of aPDI are required to provide a basis for full clinical trials. However, we believe that photodynamic inactivation should be considered an important treatment and/or prevention option worthy of further investigations.

## Data Availability

The data presented in this study are available in the article and supplementary materials.

## Supplementary Materials

The following supporting information can be downloaded at: https://www.mdpi.com/article/10.3390/antiox12040847/s1, Table S1: Relative frequencies of identified taxa abundances—file: rel_freq.xlsx.
